# Supplementary material for: MetaRibo-Seq measures translation in microbiomes
Source: Nat Commun. 2020 Jun 29;11:3268. doi: 10.1038/s41467-020-17081-z (PMC7324362; doi:10.1038/s41467-020-17081-z)
Supplement: Supplementary file 10 — Supplementary Data 7 [file 41467_2020_17081_MOESM10_ESM.zip › File2/Confidence_VeryHigh_Taxonomy/301145_out.krona.html]

Javascript must be enabled to view this page.

members
magnitude
magnitudeUnassigned
count
unassigned
taxon
rank

301145\_out

10

2
superkingdom
10

1239
9
phylum

909932
class
9

909929
9
order

1843491
family
9

970
genus
9

9
species

SRS016133\_contig\_number\_contig-100\_2349.2350SRS017227\_contig\_number\_contig-100\_612.350314SRS019792\_contig\_number\_82SRS077520\_contig\_number\_57432SRS097905\_contig\_number\_24663SRS142787\_contig\_number\_27423SRS144124\_contig\_number\_43756SRS147106\_contig\_number\_contig-100\_1437.231719SRS147547\_contig\_number\_35134
69823

976
phylum
1

200643
1
class

order
1
171549

1
family
171551

836
1
genus

1
species

SRS102808\_contig\_number\_51710
997829
